# Supplementary material for: Epidemiology and Molecular Analyses of Influenza B Viruses in Senegal from 2010 to 2019
Source: Viruses. 2022 May 16;14(5):1063. doi: 10.3390/v14051063 (PMC9143141; doi:10.3390/v14051063)

Supplementary Figure S1: Alignment of the amino acid sequences of HA gene (B/Vic) of twelve Senegalese strains that circulated in 2018 and 2019. Vaccine strain B/Brisbane/60/2008 was used as reference strains. Deletion in HA gene are indicated in the orange box.

```
[                                   1111111111 1111111111 1111111111 1111111111 1111111111 1111111111 1111111111 1111111111 1111111111 1111111112 ]
[                                   0000000001 1111111112 2222222223 3333333334 4444444445 5555555556 6666666667 7777777778 8888888889 9999999990 ]
[                                   1234567890 1234567890 1234567890 1234567890 1234567890 1234567890 1234567890 1234567890 1234567890 1234567890 ]
#B/Brisbane/60/2008|2008-04-08      KIPSARVSIL HEVRPVTSGC FPIMHDRTKI RQLPNLLRGY EHIRLSTHNV INAENAPGGP YKIGTSGSCP NITNGNGFFA TMAWAVPKND KNKTATNPLT
#B/Dakar/17/2018|2018-10-08         KIPSARVSIL HEVRPVTSGC FPIMHDRTKI RQLPNLLRGY EHVRLSTHNV INAEDAPGGP YEIGTSGSCP NITNGNGFFA TMAWAVPKN- --KTATNPLT
#B/SENEGAL/01/2018|2018-01-23       KIPSARVSIL HEVRPVTSGC FPIMHDRTKI RQLPNLLRGY EHVRLSTHNV INAEDAPGGP YKIGTSGSCP NITNGNGFFA TMAWAVPKND KNKTATNPLT
#B/SENEGAL/02/2018|2018-09-24       KIPSARVSIL HEVRPVTSGC FPIMHDRTKI RQLPNLLRGY EHVRLSTHNV INAEDAPGGP YEIGTSGSCP NITNGNGFFA TMAWAVPKN- --KTATNPLT
#B/SENEGAL/03/2018|2018-10-01       KIPSARVSIL HEVRPVTSGC FPIMHDRTKI RQLPNLLRGY EHVRLSTHNV INAEDAPGGP YEIGTSGSCP NITNGNGFFA TMAWAVPKN- --KTATNPLT
#B/SENEGAL/04/2018|2018-10-08       KIPSARVSIL HEVRPVTSGC FPIMHDRTKI RQLPNLLRGY EHVRLSTHNV INAEDAPGGP YEIGTSGSCP NITNGNGFFA TMAWAVPKN- --KTATNPLT
#B/SENEGAL/05/2018|2018-10-17       KIPSARVSIL HEVRPVTSGC FPIMHDRTKI RQLPNLLRGY EHVRLSTHNV INAEDAPGGP YEIGTSGSCP NITNGNGFFA TMAWAVPKN- --KTATNPLT
#B/SENEGAL/06/2018|2018-10-22       KIPSARVSIL HEVRPVTSGC FPIMHDRTKI RQLPNLLRGY EHIRLSTHNV INAEDAPGGP YEIGTSGSCP NITNGNGFFA TMAWAVPKN- --KTATNPLT
#B/SENEGAL/07/2018|2018-10-22       KIPSARVSIL HEVRPVTSGC FPIMHDRTKI RQLPNLLRGY EHVRLSTHNV INAENAPGRP YEIGTSGSCP NITNGNGFFA TMAWAVPKN- --KTATNPLT
#B/Dakar/02/2019|2019-01-08         KIPSARVSIL HEVRPVTSGC FPIMHDRTKI RQLPNLLRGY EHVRLSTHNV INAEDAPGRP YEIGTSGSCP NITNGNGFFA TMAWAVPKN- --KTATNPLT
#B/Dakar/06/2019|2019-02-22         KIPSARVSIL HEVRPVTSGC FPIMHDRTKI RQLPNLLRGY EHVRLSTHNV INAEDAPGRP YEIGTSGSCP NITNGNGFFA TMAWAVPKN- --KTATNPLT
#B/Dakar/08/2019|2019-03-21         KIPSARVSIL HEVRPVTSGC FPIMHDRTKI RQLPNLLRGY EHVRLSTHNV INAEDAPGRP YEIGTSGSCP NITNGNGFFA TMAWAVPKN- --KTATNPLT
#B/Dakar/07/2019|2019-03-04         KIPSARVSIL HEVRPVTSGC FPIMHDRTKI RQLPNLLRGY EHVRLSTHNV INAEDAPGRP YEIGTSGSCP NITNGNGFFA TMAWAVPKN- --KTATNPLT
```

Supplementary Figure S2. Phylodynamic tree of HA gene for Senegalese circulating strains. The tree was obtained by the Markov Chain Monte Carlo method using the BEAST software. 200,000,000 steps were required and the GTR + G model was used. Strain B/Lee/1940 was used to root the tree. The Senegalese strains are indicated in orange.

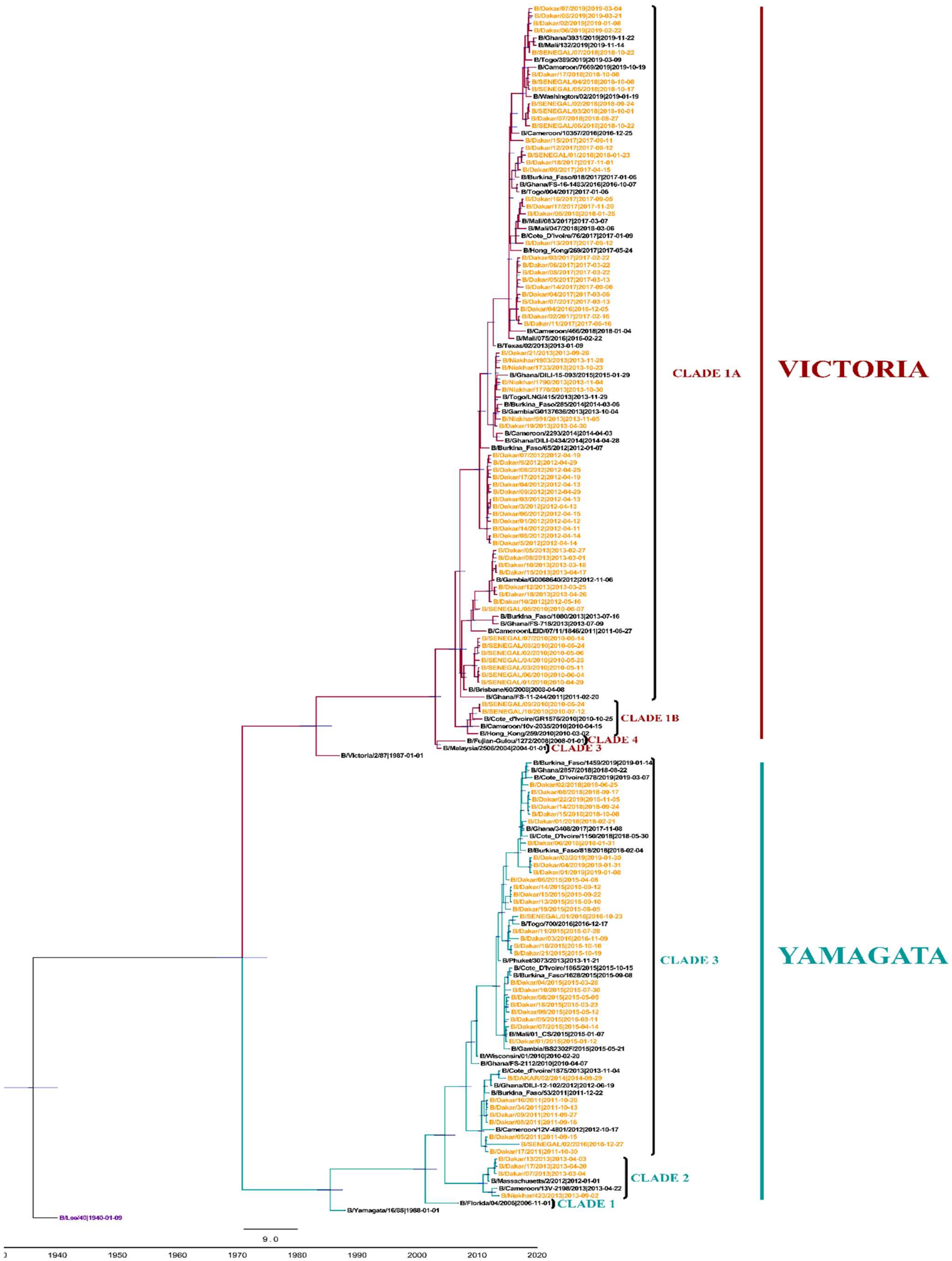

Supplementary Figure S3. Phylodynamic tree of HA gene for Senegalese circulating strains. The tree was obtained by the Markov Chain Monte Carlo using the BEAST software. 200,000,000 steps were required and the GTR + G model was used. Strain B/Lee/1940 was used to root the tree. The Senegalese strains are indicated in orange.

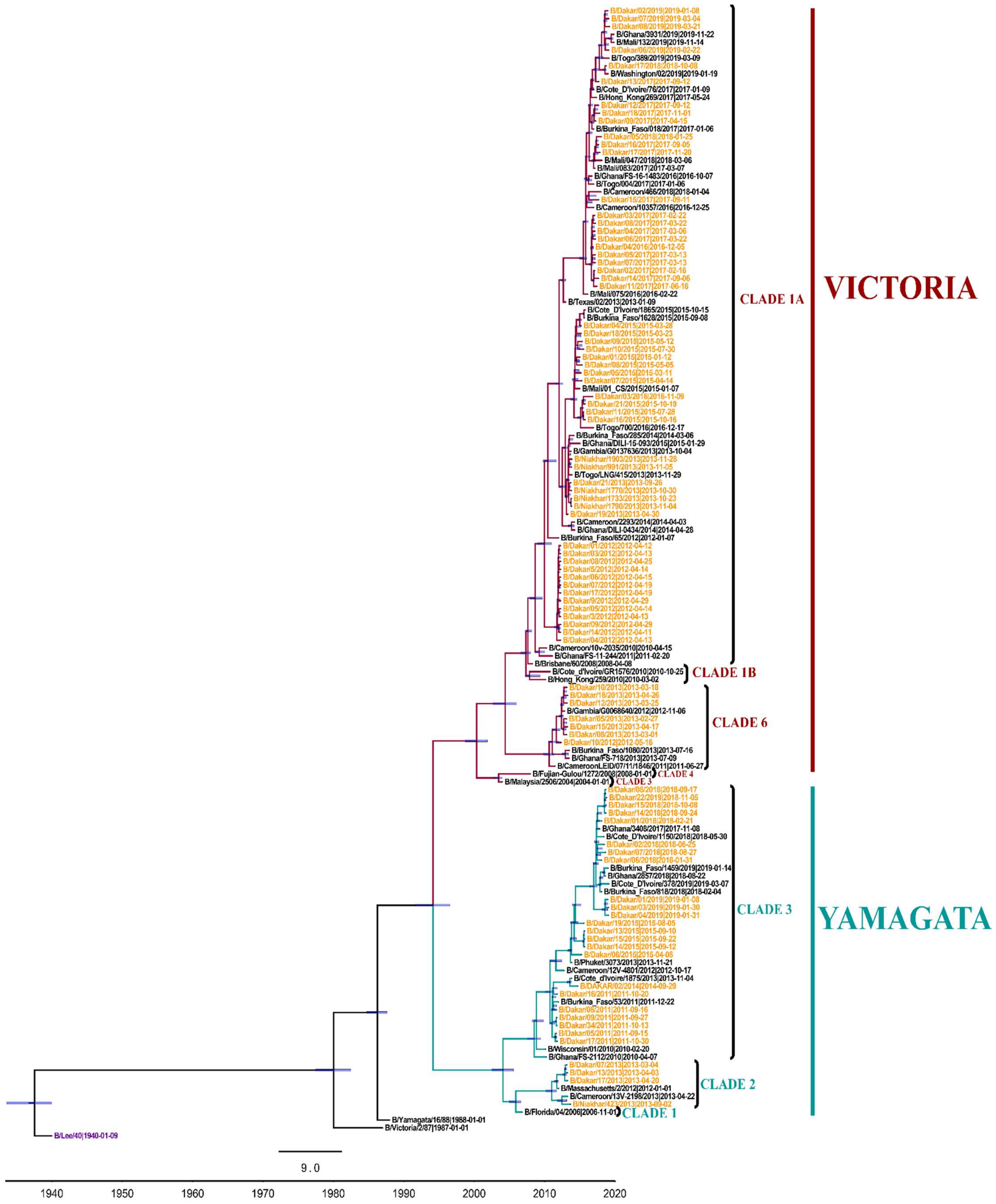

Supplement: Supplementary file 1 [file viruses-14-01063-s001.zip › viruses-1711766-supplementary.pdf]
